# Observations on the Rigidity of the Os Uteri and Perinæum

**Published:** 1856-04

**Authors:** Abraham Livezey

**Affiliations:** Lumberville, Penn.


### *Observations on Rigidity of the Os Uteri and Perinæum.* By Abraham Livezey, A. M., M. D., Lumberville, Penn.

[Communicated for the Boston Medical and Surgical Journal.]

The medical man who has had much experience in obstetrical practice must have, not infrequently, been observant of the fact, that an unusual rigidity of the os uteri and perinæum is the chief cause of protracting the agony of parturition; that, notwithstanding painful contractions of the uterus may occur every few minutes, and apparently with a degree of force sufficient for safety, yet upon repeated examinations he is mortified to find the os still undilated and undilatable, whilst he is importuned by the females in attendance upon their suffering friend, and whom he finds some difficulty in persuading that " all is right," because they cannot duly appreciate the obstacles to be overcome. In such cases, when the os uteri remains unyielding for a long time, " it is an evidence," says Dr. Dewees, " that the natural processes, which so beautifully, kindly and safely effect the change, have, from some cause or other, been interrupted." Now the question should arise in the mind of every humane accoucheur, what are the means which can be resorted to in such cases, with all confidence as to their safety and power, to restore force to those natural processes, and thus remove all difficulties in the way to a speedy parturition?

The usual routine practice in such cases is well known: a venesection, a solution of ant. et pot. tart in small doses, warm mucilaginous stupes to the perinæum sometimes, a " tumbler of warm tea taken at a draught," and then comes patience."—(Meigs,) But with what feelings of humanity can the attendant physician coolly advise *patience* to a woman well nigh exhausted by direful throes, which, perhaps, have already continued for twelve or eighteen hours, or even more? when a frail body, prostrated and helpless before him, is ever and anon *agonized* by fruitless contractile efforts of the uterus to free itself of its burden! when, with plaintive wails, *miserabile anditu*, in the tomb-like silence of the lying-in chamber, she beseeches him, at the end of every throe, to save her, lest she perish! Of adamant must be that man's heart, and wholly calloused to human suffering is he, who can sit listlessly by, with folded arms, under such circumstances, and advise patience, or say " peace, be still!" For there is no peace for that poor woman in the agony of travail; she cannot be still whilst racked by the dreadful exertions of the uterus.

What, then is the accoucheur to do? What can he do? Simply resort to some measures calculated to overcome the tension or rigidity of the parts implicated in delaying the advance of the fœtal head. But he has already tried bleeding, antimony, warm teas, extract of belladonna, pehaps, andyet hour after hour elapses with little or no perceptible change. Now when these means fail, let the accoucheur, without prejudice, resort to one other remedial agent, of the many with which Nature has so bountifully supplied us. Let him make an infusion of lobelia inflata (℈i.–ii. ad aq bull. Oj.) and inject the half or the whole, if it can be retained, into the rectum, immediately upon the subsidence of a pain. A few minutes' retention is generally sufficient to produce a marked effect. The lobelia is the most powerful relaxant in the Materia Medica, and one from which no danger need be apprehended. Its peculiar powers are speedily diffused by contiguous and continuous sympathy to the os uteri and perinæum, and the supervening pains show a manifest dilatation of the os, whilst the perinæum, if hitherto rigid, yields readily to the advancing head. Never have I been more convinced of the superior efficacy of lobelia injections, to the ordinary means, than in the attendance and delivery of several cases during the past two years, two or three of which it may be profitable to the profession to specify.

At 5 o'clock, P. M., Feb. 20th, 1854, I received a note from one of my neighboring Physicians, in which I was requested to accompany the bearer, with my forceps, for the purpose of delivering Mrs. H., a patient of his, who had been in strong labor since morning. I was informed that the head had been engaged and locked in the inferior strait for four hours without the least change or alteration—that in order to adapt it to the (supposed) contracted strait, he had, by pressure, caused the bone of the cranium to overlap, and now in her exhausted state, with a threatening of eclampsia, he wished to have her delivered at once. Upon examination, I found the head free and rotating against a very firm, rigid perinæum; and consequently some measures to produce speedy relaxation were alone indicated, for the poor woman was almost insensible from agonizing pain. The time had passed to resort to bleeding, antimonials, or to advise *patience*. Hence we prepared an enema of pulv. lobelia in flaxseed mucilage, and handing it to the nurse, stepped out of the room. In a few minutes, we were recalled, and found the crown projecting from beneath the pubal arch, and at the ensuing pain the child was wholly born. It survived but two days. At a subsequent confinement, last March, I resorted to one or two lobelia injections, much earlier in the labor, and delivery was effected in less than half the usual time and without an unpleasant symptom.

Besides the relaxation of tissues or muscular fibre, induced by this plant, and in this manner, it adds decidedly to the tenesmic force present, and thus aids in a two-fold manner in expediting parturition.

Early in the morning of the 15th of April, 1855, I was called to attend Mrs. K., a robust woman, in labor with her second child. The pains were regular and pretty strong during the whole day, and a painful, sleepless night ensued. On the following morning, finding an exceedingly rigid condition of the soft parts, and the os but slightly dilated, I took sixteen ounces of blood and put her upon the use of an antimonial solution. At 5, P. M., I called again, and found my patient much exhausted and in a continued agony of pain—of fruitless pain—for even now it was with difficulty, and not without giving her pain, that I could insert my index and middle fingers, horizontally, through the ostium-vaginæ, as she lay upon her side. But little dilatation had ensued, and an unusual mass of rigid muscles seemed to line the pelvis and guard the outlet. Exceedingly discouraged, as well as mortified, and under the impression that the poor woman would never survive the birth of the child, at least without further aid, I concluded, as a *dernier resort*, and before sending for counsel, to have enemata of infusion of lobelia administered every half hour until three was taken. Having given such orders, I withdrew to another apartment, under painful forebodings, to await the result. A short time after the third injection was given, I was recalled, and found, upon examination, that the os uteri had relapsed its rigid grasp upon the vertex of the child, and the whole crown was pressing against a soft, yielding perinæum. This woman was delivered of her first child, in England, by the aid of instruments, under the impression of a contracted or slightly deformed pelvis.

In the evening of the 20th of June, another of those tedious, distressing cases came under my care. Labor seemed to be progressing, at least so far as the regularity and force of the pains were an indication thereof; but owing to a rigid state of the os uteri, eighteen hours elapsed before a portion of the vertex, an inch and a half in diameter, was admitted through it. Further dilatation was now arrested, and this portion of the crown was grasped, as with a cord, by the patent circumference of the os with such pertinacity, that I resorted to a vensection and small doses of tartar emetic frequently repeated, which availing nought, however, I felt obliged to procure a syringe and give her enemata of lobelia infusions to alleviate her sufferings, which for the last hour had been very great; besides, her head had commenced aching violently, which of itself is always a source of alarm to the accoucheur, and demands promptness of action in instituting some means to hasten delivery. The injections, as in all previous and several like cases subsequently, proved entirely satisfactory and successful in causing the os to "open wide its mouth," in relieving the head of its pain, and the vessels of their tension. A deep indentation was observed at birth, encircling a turgid (strangulated) portion of the crown of the child's head. Such cases are not infrequent, and I now always resort to lobelia injections instead of venesections, with the happiest effect and with the promptest relief.

In conclusion, let me say that lobelia used thus in obsterical practice is a pure and safe relaxant, and where bleeding may be an *anceps remedium* on account of a weakly or debilitated habit of body (anemia), or from fear of a too great loss of blood at and after parturition for the patient's safety, it (lobelia) is unquestionably *the* remedy. It also obviates convulsions, when threatening in protracted labors from causes cited above, by its excito-relaxant powers, changing the "field of excitement" from the brain to the rectum and contiguous parts.

